# Incorporation of Hepatitis C Virus E1 and E2 Glycoproteins: The Keystones on a Peculiar Virion

**DOI:** 10.3390/v6031149

**Published:** 2014-03-11

**Authors:** Gabrielle Vieyres, Jean Dubuisson, Thomas Pietschmann

**Affiliations:** 1 Institute of Experimental Virology, Twincore, Center for Experimental and Clinical Infection Research, a Joint Venture between the Medical School Hannover and the Helmholtz Center for Infection Research, 30625 Hannover, Germany; E-Mail: thomas.pietschmann@twincore.de; 2 Institut Pasteur de Lille, Center for Infection & Immunity of Lille (CIIL), F-59019 Lille, France; E-Mail: jean.dubuisson@ibl.fr; 3 CNRS UMR8204, F-59021 Lille, France; 4 Inserm U1019, F-59019 Lille, France; 5 Université Lille Nord de France, F-59000 Lille, France

**Keywords:** hepatitis C virus, envelope, E1, E2, assembly, glycoprotein, budding

## Abstract

Hepatitis C virus (HCV) encodes two envelope glycoproteins, E1 and E2. Their structure and mode of fusion remain unknown, and so does the virion architecture. The organization of the HCV envelope shell in particular is subject to discussion as it incorporates or associates with host-derived lipoproteins, to an extent that the biophysical properties of the virion resemble more very-low-density lipoproteins than of any virus known so far. The recent development of novel cell culture systems for HCV has provided new insights on the assembly of this atypical viral particle. Hence, the extensive E1E2 characterization accomplished for the last two decades in heterologous expression systems can now be brought into the context of a productive HCV infection. This review describes the biogenesis and maturation of HCV envelope glycoproteins, as well as the interplay between viral and host factors required for their incorporation in the viral envelope, in a way that allows efficient entry into target cells and evasion of the host immune response.

## 1. Introduction

Discovered in 1989, hepatitis C virus infects 3% of the world population [1]. Associated with an 80% chronicity rate, the virus is responsible for liver disease, evolving from liver fibrosis and cirrhosis to hepatocellular carcinoma [2]. The therapy against HCV has recently taken a tremendous leap forward with the development and commercialization of directly acting antivirals. These new molecules will likely progressively replace, with the expected licensing of additional novel drugs, the interferon and ribavirin-based therapy [3]. However, the incomplete efficacy of these antivirals against the circulating spectrum of HCV variants, the emergence of viral resistance and the difficulty to treat patients with an advanced liver disease are important clinical problems that need to be addressed.

**Figure 1:**
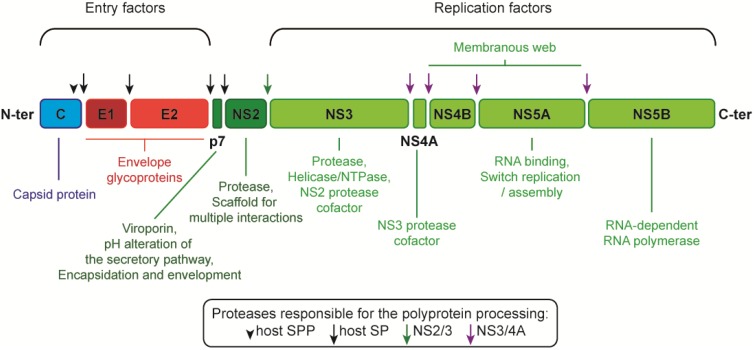
Hepatitis C virus (HCV) polyprotein. Cleavage of the polyprotein by host and viral proteases (see the symbol key) releases the mature viral proteins: the structural proteins core (C), E1 and E2 from the N-terminal third of the polyprotein, and the non-structural proteins p7, NS2-5B from the C-terminal part. SP, signal peptidase; SPP, signal peptide peptidase.

HCV belongs to the hepacivirus genus, which together with the flavivirus, pestivirus, and pegivirus genera constitutes the *Flaviviridae* family. Members of the *Flaviviridae* family share a common genomic organization. Their short positive-strand RNA genome encodes a single polyprotein, which serves as a precursor for the structural and non-structural proteins. In the case of HCV, the 3000-aminoacid-long polyprotein is cleaved into 10 mature products: the structural proteins core, E1 and E2, and the non-structural (NS) proteins p7, NS2-3-4A-4B-5A-5B (Figure 1). NS3 to 5B are necessary and sufficient for the viral RNA replication [4]. They are also involved, together with p7, NS2 and the structural proteins, in the assembly of progeny virions [5]. This extensive participation of non-structural proteins in the virion assembly process is a common feature of the *Flaviviridae* [6]. 

**Figure 2:**
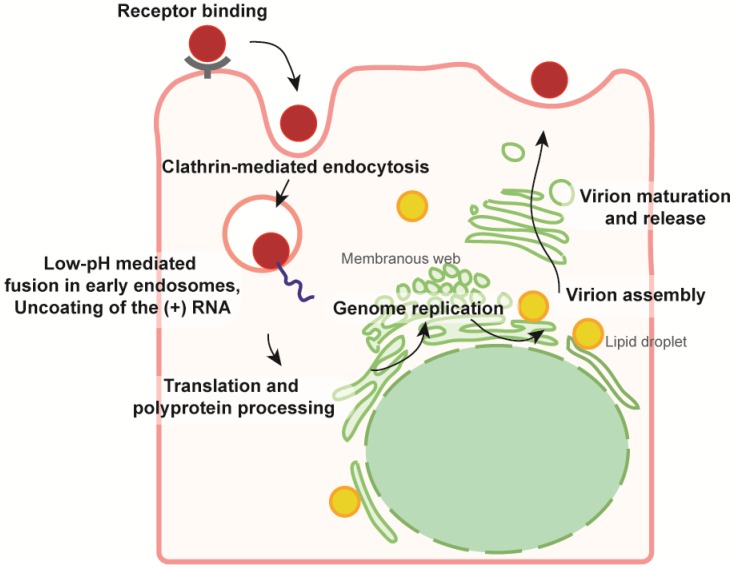
Overview of HCV replication cycle in hepatocytes. Note that this schema is an extremely simplified representation. The viral particle is represented by a red disk. Viral entry involves a long list of receptors, it occurs by clathrin-mediated endocytosis and terminates with the low-pH induced fusion between viral and endosomal membranes [7]. The released genome is translated into a single polyprotein, which is processed by host and viral proteases into the individual mature proteins. Replication of the viral RNA occurs in vesicles formed by modified ER membranes [8]. Assembly of new virions (see Figure 6) also occurs at the ER membrane, but in close proximity to lipid droplets (LDs). Viral capsids bud within the ER lumen, travel through the Golgi apparatus and are released via the secretory pathway. More detailed pictures of the individual steps are available in the reviews cited above.

The HCV replication cycle is summarized in Figure 2. Briefly, HCV binds and enters the cell in a multistep process involving numerous host receptors and co-receptors (for a review on HCV entry, see for instance [7]). Internalization occurs via clathrin-mediated endocytosis, which guides the virion towards early endosomes, where fusion is triggered by pH acidification. Once in the cytosol, the genomic RNA is translated into a polyprotein. The mature gene products carry out RNA replication, in association with ER- (endoplasmic reticulum) derived membranes, and the assembly of progeny virions, which travel through the secretory pathway before secretion and infection of naive cells. 

E1 and E2, the two HCV envelope glycoproteins, have been studied since the beginning of HCV research, but investigations were restricted at first to heterologous expression systems. Their ectopic expression into mammalian cells and incorporation into retroviral HCV pseudoparticles (HCVpp) has led to our understanding of the glycoprotein synthesis, folding, topological and functional organization. The mode and characteristics of their incorporation onto the infectious virion remain however incompletely understood. Converging lines of evidence indicate that HCV glycoproteins are structurally different from the flavivirus homologs. E1 and E2 are highly glycosylated [9] and do not depend on a cleavage for their activation [10]. Moreover, despite its low-pH-triggered fusion [11,12], the hepatitis C virion is resistant to low pH in solution [13]. Finally, the pleomorphic nature of the virus particles [14,15,16], due to the lipoprotein association, also distinguishes HCV from the related flaviviruses. So many peculiarities suggest that flavivirus entry and assembly mechanisms probably do not apply to HCV. This review summarizes our current understanding of HCV glycoprotein incorporation into the viral envelope, from their biogenesis in the ER until their exposure onto the virion surface as functional entry units. 

## 2. From the Glycoprotein Assembly-Line to a Functional Glycoprotein Heterodimer

### 2.1. Translation and Cleavage of E1E2 from the Polyprotein Precursor

As for many RNA viruses, HCV proteins are synthesized sequentially from a common polyprotein precursor (Figure 1). The structural proteins are cleaved from the N-terminal moiety of this polyprotein and the two envelope glycoproteins, E1 and E2, are found in tandem in this region. Translation of E1 signal peptide directs the translation machinery and nascent polypeptide to the ER and the translocon complex ensures that the rest of the polyprotein adopts the appropriate topology on either side of the ER membrane. The envelope glycoproteins, as well as the p7/NS2 junction, are cleaved from this precursor by the ER signal peptidase complex, whereas an ER intramembrane-cleaving signal peptide peptidase (SPP) releases the E1 signal peptide from the core protein. The signal peptides of E2 and p7 remain attached to the upstream proteins (E1 and E2, respectively) as signal anchor sequence, thus forming an integral part of the E1E2 transmembrane domains (TMD). Thus, the E2 signal peptide corresponds to the E1 C-terminus and the p7 signal peptide to the E2 C-terminus. 

Interestingly, cleavages at the E2/p7 and p7/NS2 junctions are delayed so that both E2-p7 and E2-p7-NS2 precursors can be detected in infected cells [17]. These uncleaved precursors are dispensable for HCV replication cycle as shown by the dissociation of E2, p7 and NS2 expression in bicistronic constructs [18]. Moreover, E2-containing precursors are not detected in the released virus particles [10]. A timely processing at the E2/p7 junction site is however important for efficient virus production [19]. How the fine-tuned proteolytic cleavage at these sites facilitates virus production is not clear. It is possible that the timely maturation of the polyprotein assists folding and/or oligomerization of the involved proteins or helps to liberate distinct protein functions at the proper site, dose or timing.

**Figure 3:**
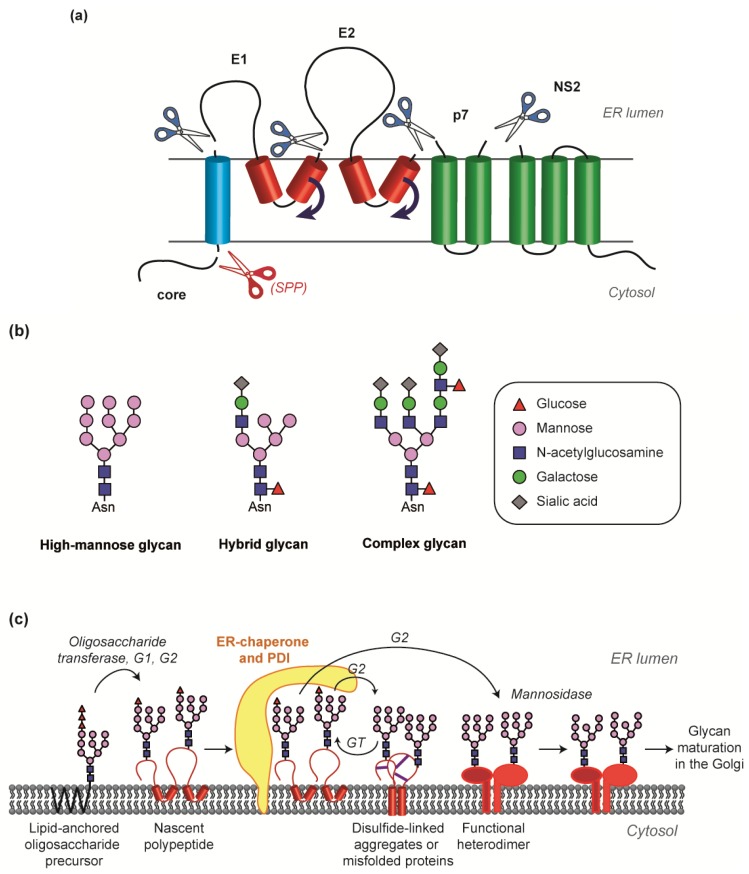
Folding and maturation of E1 and E2 envelope glycoproteins. (**a**) Flipping of E1 and E2 transmembrane domains (TMD) upon cleavage of the polyprotein. (**b**) Different types of N-glycans. High-mannose glycans are generated in the ER lumen and can be further modified in the Golgi apparatus into hybrid or complex glycans. In the HCVcc system, intracellular E1E2 harbor high-mannose type glycans whereas some of the virion-associated E1E2 glycans are modified into hybrid or complex glycans [10]. (**c**) E1E2 folding is concomitant with glycosylation and disulfide bond formation. The picture illustrates E1E2 glycosylation in the ER. Oligosaccharide precursors are transferred from their lipid anchor onto the nascent polyprotein chain, and terminal glucose residues are trimmed by glucosidases (G1, G2, glucosidases 1 and 2). The resulting glycan intermediates are recognized and folded by ER chaperones, which associate with protein disulfide isomerases (PDI). Misfolded proteins undergo cycles of deglucosylation (G1, G2) and reglucosylation (GT, glucosyltransferase) until the proper folding is achieved. The chaperone-PDI complex then releases the glycoprotein, which can undergo further glycan trimming and maturation in the Golgi apparatus. Purple lines represent intermolecular disulfide bonds.

Both E1 and E2 glycoproteins are type I transmembrane proteins with a large N-terminal ectodomain facing the ER lumen and a C-terminal single-membrane spanning transmembrane domain (TMD) [20]. Curiously this topology is counter-intuitive as one would rather expect the glycoprotein TMDs to form hairpins due to their dual function as stop transfer sequence and signal sequence (see Figure 3). Consistent with this, E1 and E2 TMDs consist of two hydrophobic stretches separated by one or several conserved polar residues, which could form a short cytosolic loop or may, at least transiently, stabilize a hairpin-like conformation with possible functional relevance. However, the hydrophobic stretches are abnormally short for membrane-spanning segments, suggesting that a hairpin topology, if existing, is only transient. This dilemma was solved by the recognition of the flexibility of E1 and E2 TMDs [21]. In brief, upon polyprotein translation, E1 and E2 TMDs form a hairpin, with both hydrophobic stretches extended to cross the ER lumen and the hydrophilic residues exposed to the cytosol, or buried in the translocon complex. Cleavage of the glycoproteins releases the constraint imposed on E1 and E2 TMDs, whose second hydrophobic stretch flips and forms together with the first stretch a single membrane-spanning segment, oriented from the lumen to the cytosol. Although changes in topology of viral envelope proteins have been reported for other viruses (for instance in the case of HBV [22]), to our knowledge, this is the only demonstration for the specific reorientation of a portion of a TM segment in a viral glycoprotein.

### 2.2. The E1E2 Tandem: Determinants for Heterodimerization

Although the oligomeric status of the global HCV envelope protein complex might fluctuate during the HCV replication cycle [10,23], in particular at the envelopment and fusion stages, the E1E2 heterodimer seems to be a stable basic entity [10,24,25,26]. The expression of E1 and E2 in tandem within the viral glycoprotein predicts a common fate for the two glycoproteins. Coevolution of the two protein sequences emphasizes the importance of their interaction [27,28,29,30]. The expression of E1 and E2 from a common precursor is however not required for E1E2 heterodimerization. Indeed, even if expressed in *trans* and in the absence of any other HCV protein, the two proteins heterodimerize in the ER [31,32]). Also, glycoproteins expressed separately in *trans* heterodimerize and are incorporated in the virion in the context of the HCVpp [33] and *trans*-complementated HCV particles (HCV_TCP_) [34]. This interaction between E1 and E2 relies on non-covalent bonds [31,35]. Interestingly, a fraction of E1E2 complexes as well as some E1 dimers and trimers are resistant to SDS and can be observed in SDS-PAGE [10,23,36,37].

The main heterodimerization interface is composed by E1 and E2 TMDs [38,39,40,41,42,43,44]. Their truncation or replacement by homologous regions of the flavivirus envelope proteins abolishes heterodimerization [45]. More precisely, mutation of the charged residues located in the middle of E1 and E2 TMDs abrogates the heterodimer formation [32,44]. Moreover, mutations within the N-terminus of E1 TMD also abolish heterodimerization [43,44]. Contrary to the TMDs, the glycoproteins ectodomains alone are not able to heterodimerize; however, they participate in the process [27,46,47,48]. In particular, as already observed with the homologous region of the flavivirus E protein, the E2 stem region (directly following the TMD) and its conserved heptad repeat are crucial for E1E2 heterodimerization [46]. Finally, deletion of the hypervariable region 2 (HVR2) or the intergenotypic variable region (IgVR) also abrogates E1E2 complex formation [47].

### 2.3. Chaperone-Mediated Folding, Disulfide Bond Formation and Glycan Maturation

Noteworthy, in heterologous expression systems, a fraction of intracellular E1E2 proteins form non‑functional disulfide-linked aggregates. These can be discriminated from the E1E2 heterodimers by their association with ER chaperones, their sensitivity to protease digestion, their non-reactivity with specific conformation-sensitive antibodies or by SDS-PAGE in non-reducing conditions [24,35,49]. This tendency to aggregate reflects the long and complex E1E2 folding process. Generally, protein folding occurs in the ER and involves a number of enzymes including members of the protein disulfide isomerase (PDI) family that catalyze the formation and isomerisation of disulfide bridges, peptidyl prolyl isomerases and components of the N-glycosylation system. With their abundant N-glycans and disulfide bonds, HCV glycoproteins nicely illustrate the complexity of protein folding. 

While the presence of O-glycans on HCV envelope glycoprotein remains disputed, their N‑glycosylation has been extensively studied [50,51,52,53]. Actually, N-glycans account for half the mass of HCV glycoproteins, with up to 5 glycans on E1 and up to 11 on E2 (genotype 1a). They modulate HCV infectivity and neutralization but also determine the folding and heterodimerization of E1E2 [50,53]. N-glycosylation starts in the ER. The sequence of events leading to high-mannose, hybrid or complex glycans has been described in detail by others [54]. In brief, a core oligosaccharide is formed in the ER and transferred *en bloc* by an oligosaccharyltransferase from its dolichol anchor onto the nascent polypeptide. A complex sequence of glycan trimming, protein folding and glycan modification follows and can continue in the Golgi apparatus. Hence, N-glycosylation offers a wide range of variations on the basis of a core oligosaccharide [54] (Figure 3b). In the ER, the variability is limited to high-mannose glycans. However, proteins that traffic to the Golgi display a much broader variety of hybrid or complex glycans that result from the transfer by Golgi transferases of galactose, N‑acetylglucosamine, sialic acid or fucose residues onto the glycan precursor. This is the case for HCV glycoproteins that travel through the Golgi incorporated in the viral particle [10,55]. 

Glycosylation is tightly associated with protein folding [54]. Indeed, the quality of protein folding is controlled by chaperones that assess and assist folding, retain misfolded proteins in the ER and eventually target them for degradation. Calnexin and calreticulin are N-glycoprotein-specific chaperones that associate with E1E2 [56,57]. In fact, they contain a lectin site that recognizes an early ER glycosylation intermediate, and a binding site for ERp57, a member of the PDI family. Therefore, glycoprotein intermediates are recognized by the calnexin and/or calreticulin chaperones, which recruit the ERp57 isomerase. ERp57 then catalyzes the formation of disulfide bridges between cysteine residues. This multiprotein complex dissociates and re-associates on the glycoprotein until it is properly folded or degraded. When folding is accurate, the protein is released by the quality control machinery and can finally be exported to its final destination. This folding and quality control processes are summarized in Figure 3c. 

Mismatching of E1E2 cysteines is responsible for the covalently linked aggregates observed in heterologous expression systems [24,35,49]. In the functional glycoproteins, the cysteines are linked by intramolecular disulfide bridges and E1E2 form non-covalent heterodimers. The disulfide network is rather flexible in E1, where mutation of individual cysteine residues only attenuates the virus [58]. In contrast, mutation of E2 cysteine residues, individually or in pairs, is lethal [59]. To ensure the correct connectivity of their numerous cysteine residues, E1 and E2 must fold slowly and cooperatively (reviewed in [60]). This is not only supported by the ER chaperones, but also by the reciprocal chaperoning between E1 and E2 [40,41,61]. This contrasts with flaviviruses where only the prM companion protein, believed to be E1 homolog, assists the folding of the fusion protein. 

### 2.4. Glimpses into E1 and E2 Structures and Shift in Fusion Paradigms

So far, both the crystal structure of the HCV glycoprotein complex and the 3D reconstruction of the virion are missing. The heterogeneity of the virions, the difficult to purify them and their resemblance to VLDL limit the resolution and precision of structural information obtained by cryoelectron microscopy [14,15,16] (see Section 3.1). Efforts to crystallize E1 and E2 have also met several challenges inherent to the biology of the glycoproteins, including the presence of transmembrane domains, of a dense glycan shield and the necessary cooperation between E1 and E2 for their folding. In the absence of crystallographic data, related viral glycoproteins served as a scaffold to build homology-based structural models. Briefly, E1 or E2 protein sequence was aligned to the envelope protein of closely related viruses with a known structure, typically their fusion protein. However, in the case of HCV, the identity of the fusion protein remains unknown, so it is not clear which one of E1 or E2 protein should be aligned to the flavivirus E protein for instance. Computational analysis was applied to obtain a 3D model. This model was then further refined to accommodate the experimental data (for instance the exposure of the receptor binding sites, glycosylation sites and antibody epitopes, the heterodimerization surface, putative fusion peptide, and more recently, the disulfide bridge connections [62]). 

Surprisingly however, both E1 and E2 models have been calculated based on their homology with the same protein, namely the TBEV envelope protein E [63,64]. In both cases, the authors found a homology resulting in the description of either E1 [64] or E2 [63] as a class II fusion protein. Also, the small size of E1 and E2 ectodomains as compared to their flavivirus parent (E ectodomain) is startling. This suggested that HCV might have a truncated class II fusion protein [64,65] or that both glycoproteins might participate in the fusion process, as also suggested by E1E2 mutagenesis and functional fusion assays [29,66,67,68,69,70]. Interestingly, similar analyses successfully predicted the bunyavirus class II fold and fusion loop [71,72].

More recently, Krey *et al.* proposed a more refined model for E2 ectodomain [62]. The authors gathered new experimental data on the disulfide connectivity between the conserved cysteine residues of soluble E2 produced in *Drosophila* cells. They integrated this data together with the experimentally determined and computationally predicted secondary structure of E2 to thread the E2 sequence onto the scaffold of a class II fusion protein, typically an alphavirus or flavivirus envelope protein. This structure encompasses the 3 domains characteristic of the class II fusion proteins. Domain I is positioned at the center of the structure and forms a beta-barrel with insertions corresponding to domain II, which carries the fusion peptide. Domain III is linked to domain I by a flexible linker allowing its translocation during fusion, and exhibits an immunoglobulin superfamily fold. It is also attached to the TMD via the flexible stem region [73]. This model pointed at a potential fusion peptide in E2 and proved useful to study E2 function (see for instance [27,28,47]).

Noteworthy, all these studies were based on the postulate that HCV had a class II fusion protein, similarly to alpha- and flaviviruses [74]. This hypothesis is indeed supported by the global genomic organization of HCV, characteristic of the *Flaviviridae*, a virus family whose every single crystallized fusion protein until 2013 belonged to the class II [73]. More specifically, the synthesis of HCV glycoproteins in tandem, with the companion protein upstream, is reminiscent of the alpha- and flaviviruses, but also of the *Bunyaviridae*, a virus family whose glycoprotein was recently ascribed to the class II fusion fold [75]. Lastly, the identification of a similar fusion protein across the virus phylogenetic tree, despite the absence of sequence similarity, strengthened the postulate that HCV was using the same fusion mechanism. 

**Figure 4:**
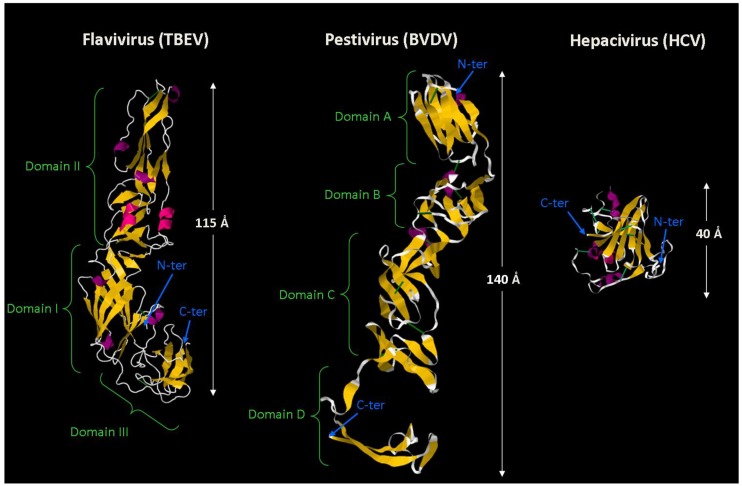
Crystal structures of the major envelope protein in the *Flaviviridae* family. A representative for each major genus of the *Flaviviridae* family is shown: TBEV E protein for the flaviviruses (PDB ID 1SVB [82]), BVDV E2 protein for the pestiviruses (PDB ID 2YQ2 [76]) and finally HCV E2 protein for the hepaciviruses (PDB ID 4MWF [78]). In each case, a monomer is represented on scale. The color code emphasizes the secondary structures with β-strands in yellow, helices in pink and purple, and flexible regions in grey. Disulfide bridges are depicted in green. Although TBEV E protein is a prototype class II fusion protein, the pestiviral and HCV E2 proteins do not comply to these rules. Note that the elongated structures of the flavivirus E and pestivirus E2 are believed to lay flat, parallel to the virion surface. TBEV E2 aminoacid chain is folded as a hairpin into three domains, domain II containing the fusion peptide; on the contrary, BVDV E2 aminoacid sequence is arranged linearly from the N- to the C-terminus into 4 domains [76,77]. E2 shows an even more divergent organization with a compact and globular structure. The arrangement of BVDV and HCV E2 proteins is distinct from any viral fusion protein described so far, suggesting that E1 might actually be the fusion protein. Note that each protein has been modified to different extents in order to facilitate its crystallization. In particular, the membrane anchor, and in the case of HCV E2 protein, the variable regions have been deleted. A comprehensive description of the exact recombinant proteins crystallized can be found in the original publications.

However, recent arguments have now challenged this hypothesis. First, HCV virion morphology does not comply with the criteria predicting a class II fusion mechanism. Indeed, HCV particles are pleomorphic with no clear outer glycoprotein scaffold [14,15,16] whereas class II viruses are typically regular enveloped viruses; that is, they exhibit a well-ordered and symmetrical glycoprotein shell covering the lipid bilayer around the nucleocapsid [73]. Secondly, no cleavage of E1, the presumed companion protein [61], has been reported during virus maturation [10] or entry. Moreover, the recent reports, by two independent groups [76,77], of the structure of the pestivirus major envelope glycoprotein (E2) cast further doubt on the previously accepted HCV fusion model. Both studies show that BVDV E2 protein is an elongated molecule consisting of four β-sandwich domains (A to D) arranged linearly from the N- to the C-terminus. This structure is distinct from a class II fold and from any known fusion protein, suggesting that the pestivirus E1 might be the actual fusion protein or that E1 and E2 act in concert. The fact that viruses, within the *Flaviviridae* family, and closer to HCV than the flaviviruses, have a different fusion strategy questioned the previous postulate. Finally, even more recently, Kong *et al.* solved the crystal structure of E2 glycoprotein core domain in complex with a neutralizing antibody [78]. Noteworthy, the protein expressed in 293T cells had to be modified to facilitate its crystallization. In particular, the variable regions and two glycosylation sites were deleted, leaving a core domain that retains the protein capacity to bind the CD81 receptor and conformation-sensitive antibodies, and to neutralize HCVcc infection. Like class II fusion proteins, HCV E2 contains a central immunoglobulin-fold β domain. Apart from this feature, E2 structure clearly does not comply with the class II criteria and also does not resemble its pestivirus homolog (Figure 4). Rather than an elongated shape, E2 forms a compact globular structure distinct from any known viral fusion protein. Interestingly, the cysteine connectivity is very different from what was reported before by Krey *et al.* [62], with only one common disulfide bond. Moreover, the masking of neutralizing epitopes by glycans and the definition of the CD81 binding site in this new structure confirms previous experimental results [79,80,81]. Altogether, these new data bring E1 on the spotlight as the new HCV fusion protein candidate. Now that this long-awaited structural insight into E2 protein is available, efforts will probably be concentrated on the determination of E1 structure and of the virion-exposed E1E2 complex, and ultimately to break the code of HCV fusion. 

### 2.5. From the ER Membrane to the Surface of Secreted Viral Particles: Viral Glycoproteins on the Way Out

Immediately after the polyprotein translation, all HCV proteins are associated, directly or indirectly with the ER membrane via diverse membrane anchors [83]. E1 and E2 are retained in the ER of infected cells via ER-localization signals in their TMDs [38,39]. This retention was demonstrated by confocal microscopy. Moreover, the presence of N-glycans on the glycoprotein ectodomain attests the glycoprotein orientation towards the ER lumen [26]. In HCV-producing cells, E2 colocalizes partially with NS3 and strongly with p7 [26,84]. Electron microscopy and confocal immunofluorescence studies also showed that at least a subset of HCV glycoproteins localizes in close proximity to core‑loaded LDs [85,86]. 

The dynamics of virus assembly is challenging to track. New developments of live-cell-compatible detection methods for HCV core protein (tetracysteine tag [86,87]), RNA [86,88] and host assembly cofactors (e.g., apoE-GFP [87]) gave a first glimpse into this issue. Unfortunately, to date, the lack of appropriate tools compatible for E1 and E2 tracking in live cells has precluded the dynamic imaging of HCV envelopment and secretion. Indirect evidence indicates however that E1 and E2 traffic through the secretory pathway during virus assembly and release. First of all, Brefeldin A, an inhibitor of the ER-Golgi transport, induces the intracellular accumulation of infectious HCV particles [55]. Furthermore, the presence of complex glycans on virus-associated glycoproteins [10], a hallmark for proteins that have transited through the Golgi apparatus, lends further support. Interestingly, the fact that only a subset of E2 glycans and no E1 glycan are modified at the HCVcc surface suggests that glycoproteins are poorly accessible to the sugar-modifying Golgi enzymes, probably because they are tightly packed at the virion surface. At the HCVpp surface however, most glycans are modified [36,37]. This discrepancy may reflect that, contrary to HCVpp, which are assembled in a post-Golgi compartment (within the cell or at the plasma membrane [33,89,90,91], HCVcc morphogenesis and envelopment occur earlier, already in the ER. Rare electron microscopy pictures [85] support this notion of HCV budding in the ER, in close proximity to LDs. 

New tagged versions of E1 or E2 proteins, compatible with live-cell microscopy, need to be developed to get a dynamic insight into HCV envelopment and release. Ideally, tagging E1 or E2 proteins could be combined with the existing core, HCV RNA or apoE tracking systems to follow the gathering of the pieces of the puzzle for HCV assembly and release. The development of super resolution fluorescence microscopy, with approaches already validated on other small animal viruses such as HIV [92], might also shed a new light on HCV assembly, budding and egress.

### 2.6. Systems to Study HCV Envelope Glycoproteins and Their Incorporation in the Virion

Systems to study HCV assembly and glycoprotein functions have been reviewed in details elsewhere [93,94]. Briefly, the main systems to study E1 and E2 glycoproteins on a viral scaffold are retroviral HCVpp, HCV_TCP_ and HCVcc. The HCVpp system relies on the incorporation of HCV envelope glycoproteins on a retroviral backbone, containing a retroviral mini-genome that encodes a reporter protein [33,89,90]. It uses the ability of retroviruses to incorporate a variety of viral and cellular glycoproteins in their envelope, since their budding is mediated by the capsid rather than the envelope [95]. This system therefore allows for the incorporation of E1E2 from a variety of clinical isolates [96]. However HCVpp assembly follows the retroviral program. As a consequence, the virion gets wrapped into a post-Golgi host membrane, does not associate with lipoproteins and harbors E1E2 glycoproteins with biased glycosylation and oligomerization patterns as compared to HCVcc [10,36]. Nevertheless, HCVpp are useful tools to specifically discriminate between entry and assembly phenotypes in the case of particular virus glycoprotein mutants or host cell perturbations.

Since 2005, HCVcc are the best compromise between robustness and authenticity [97]. Indeed, HCVcc are obtained by transfection of a full-length HCV genome in permissive cells, typically hepatoma cell lines [98]. Optimization of the system with virus chimeras and selected cell lines enabled the recovery of high-titer virus preparations [94], amenable for purification and molecular biology investigations of the HCV whole replication cycle. HCVcc recapitulate at best the properties of genuine HCV particles, albeit modulated by the specific properties of the host cell system, which does not entirely reflect the patient hepatocyte [99,100]. The differences in virion composition between HCVcc and serum-derived particles, as well as alternative systems to grow HCV particles have been reviewed elsewhere [93,101].

Finally, the HCV_TCP_ model is a variation of the HCVcc system, particularly convenient to look at HCV assembly and entry [102,103,104,105,106,107,108]. It relies on the isolation of a self‑replicating module in the HCV genome (subgenomic replicon), and on the supply in *trans* of the remaining viral proteins, which are necessary to enable the production of a viral progeny. The proteins provided in *trans* are expressed independently of the HCV subgenome replication, and therefore, one can genetically manipulate their genes, while avoiding potential side effects on HCV RNA replication and polyprotein processing. HCV_TCP_ are single-round infectious particles. They are useful tools to investigate E1E2 incorporation in an authentic viral particle, but also to generate authentic virions [104] harboring E1E2 proteins derived from clinical isolates [109]. 

## 3. HCV Envelopment and Glycoprotein Acquisition

### 3.1. HCV Virions, a Heterogeneous Population

A striking and unique feature of HCV biology is the virion association with lipoproteins. The unusually low density of serum-derived particles was the first hint for this interaction (for a comparison of HCV density to related viruses, see [7]). HCV density certainly depends on the patient and method of analysis. However, distinct viral populations are usually observed in patient sera. A very low-density fraction (<1.06 g/mL) is associated with LDL or VLDL and harbors apoB [110]. An intermediate population (1.06 to 1.21 g/mL) is combined with high-density lipoproteins (HDL). Finally, a dense population (>1.21 g/mL) comprises naked capsids and, in immunocompetent patients, virions cross-linked by antibodies [111,112,113,114,115]. 

The infectious density profile of HCVcc is also broad and shifted towards light fractions as compared to the peak of RNA and core [10,16,116,117,118]. Interestingly, glycoproteins are mostly incorporated in the infectious fractions while hardly detectable in the dense poorly infectious core- and RNA-positive fractions [10,16]. The peak of infectivity also cosediments with VLDL marker [16,118]. In particular, HCVcc incorporate apoE [14,16,118,119] and apoCI [120]. Other apolipoproteins such as apoB are present in serum-derived HCV particles [110,121,122] but dispensable for HCVcc assembly [119,123,124] (see Section 3.5). 

**Figure 5:**
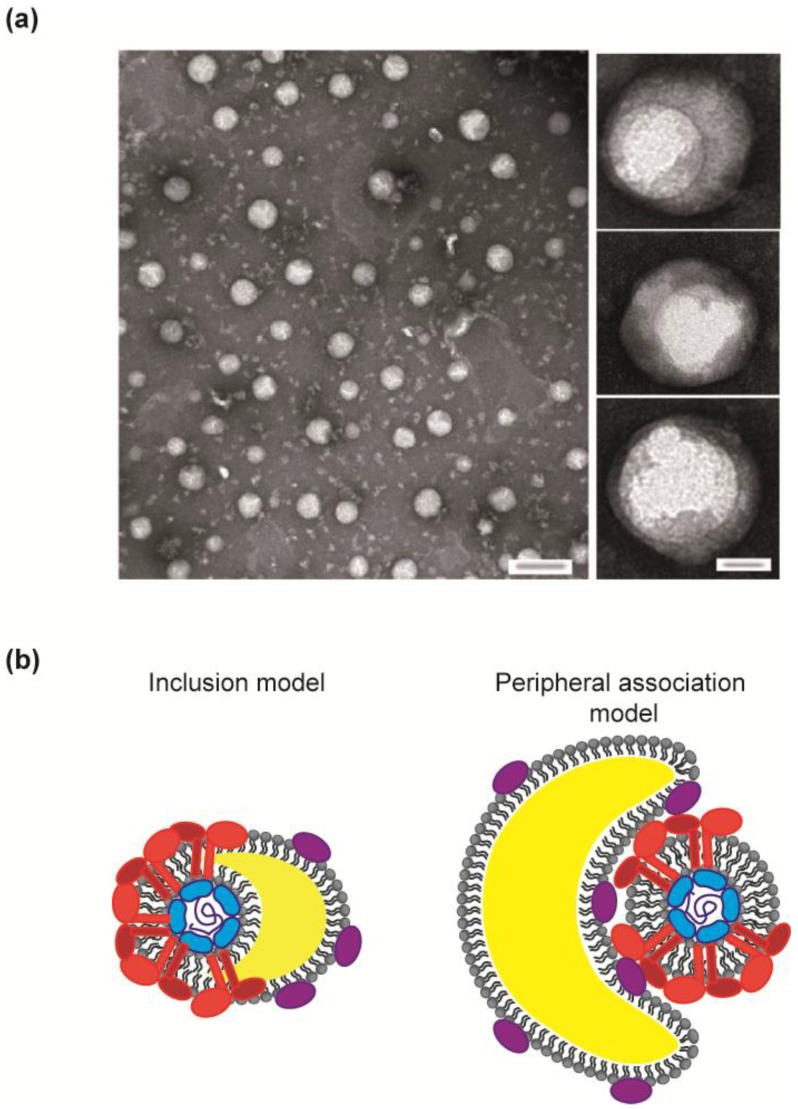
HCV virion heterogeneity. (**a**) Transmission EM pictures of HCV virions, reproduced from Catanese *et al.* [14], with permission. HCV particles were purified over heparin column and the high infectivity fractions isolated by density gradient ultracentrifugation. Note the diversity in virion size and the absence of apparent symmetry. The close-up pictures represent examples of particles with possible internal structures; although the 2D imaging technique used cannot exclude the superposition of separate particles. (**b**) Theoretical models for the HCV lipoviroparticle. Lipoprotein constituents might be incorporated within the HCV particle, thus forming a true lipoviroparticle (left panel), as supported by André *et al.* [101,110] or peripherally associated, as already proposed by Lindenbach *et al.* [5]. Please see the main text for the detailed description.

Purified infectious HCVcc have been observed by electron microscopy, eventually combined with immunogold labeling [14,15,16] (Figure 5a). These studies confirm the pleomorphic nature of HCV particles and show virions with a rather smooth and even surface [14]. Remarkably, some pictures display a spherical internal structure asymmetrically positioned within the purified virus particle (see Figure 5a, close-up pictures) [14]. 

These intriguing observations closely resemble the recently proposed structural model of HCV lipoviroparticle [101]. Such true hybrid virus/lipoprotein particles had previously been proposed by André *et al.* and supported by electron microscopy observation of patient-derived serum samples [110]. In this model, excess of neutral lipids and accompanying lipoproteins incorporated into HCV particles may cause a dilatation and swelling of a portion of the membrane bilayer, which may result in the observed asymmetric particle organization (see Figure 5b, left panel). This raises an obvious question regarding the topology of the E1E2 complex and their TMDs, which may be insufficient in length to span such a dilated bilayer. As a consequence the E1E2 complexes would be enriched at the “thin” side of the viral membrane bilayer, or the comparatively lengthy TMDs of E1E2 proteins may be an adaptation to span a lipid-enriched membrane bilayer. 

Alternatively, lipoproteins might be peripherally associated, transiently or permanently, with the canonical viral particles via interaction between apolipoproteins and HCV envelope lipids or proteins (Figure 5b, right panel). Such a model was recently proposed by Lindenbach *et al.* [5]. It is of course also possible that both types of virions co-exist. In both particle types, the interaction with lipoproteins could shield HCV glycoproteins from the host antibody neutralizing response and explain the poor detection of HCV glycoproteins at the virion surface [14,16]. Moreover, these proteins are known to bind lipoprotein receptors expressed on liver cells and therefore could facilitate homing of the HCV particle to its preferred host cells. 

### 3.2. Non-Structural Proteins Bring Together the Bricks for Assembly

**Figure 6:**
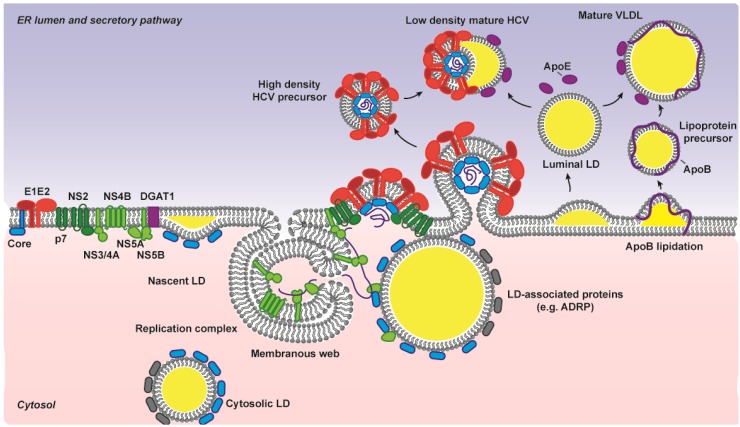
HCV assembly involves a concerted action of all viral proteins and hijacks the host lipoprotein synthesis pathway. The color code for the viral proteins is similar to Figure 1, with non-structural proteins in green, and components of the replication complex in light green. Upon cleavage of its C-terminus, core protein is loaded onto LDs, a process controlled by DGAT1 [129]. The junction between core-loaded LDs and the replication-complex-rich ER membranes forms the platform for virion assembly. Newly replicated viral genomes are transferred to the assembly sites via NS3/4A or NS5A. NS2 and p7 connect replication complexes and core proteins to the glycoproteins. Virus budding occurs by encapsidation of the viral RNA, and envelopment in the ER membrane. The subsequent interplay with the host lipoprotein pathway remains poorly understood. Note that this schema represents one hypothetical model among several. Indeed, lipoprotein components might be incorporated within the viral particle concomitant with budding, as suggested by the co-trafficking of core and apoE [87]. Alternatively, as depicted in this model, dense intracellular virions might bud and, in a second step, mature by incorporating lipoprotein components within the lumen of the secretory pathway. This could happen by acquisition of apoE and fusion with luminal LDs, normally involved in the maturation of the VLDL precursors. The differences in biophysical properties of intra- and extracellular virions support this hypothesis of a late association [117].

Another interesting feature of HCV assembly, shared with the other members of the *Flaviviridae* family, is the involvement of non-structural proteins as co-factors for infectious virus production [6]. Every single HCV protein has now been linked to HCV assembly [5]. They participate in a very concerted process involving viral RNA encapsidation, capsid envelopment, budding and association with lipoproteins (Figure 6). A more systematic description of the role of each individual viral protein in HCV assembly and release has been reviewed by others (see for instance the recent review by Lindenbach, B.D. [5]). The two main coordinators of HCV assembly are probably p7 and NS2 [17,18,125,126,127,128]. They are also of particular interest for the present topic as they link assembly and replication complexes, in other words structural and non-structural components of the replication cycle. 

The first task for HCV assembly is to gather the virion components. These include the three structural proteins, C, E1 and E2, and the viral RNA. Lipoprotein components are also incorporated into the virion (lipids and apoE, in particular), but this will be dealt with in a different paragraph. As mentioned before, E1 and E2 are ER-resident proteins. The replication complexes, and therefore the nascent RNA, are also associated with rearranged ER membranes, often forming double-membrane vesicles protruding from the ER surface [130]. However, core protein, once matured by SP and SPP cleavage, is transferred from the ER to the LD surface. This relocation is controlled by diacylglycerol acyltransferase-1 (DGAT1), an ER-resident enzyme involved in triglyceride synthesis and LD biogenesis. Via its interaction with core, DGAT1 drags the nucleocapsid proteins along the ER surface and onto nascent LDs [129]. From a first nucleation point, core can load part or the whole LD surface, by progressively displacing adipose differentiation-related protein (ADRP) [86,131]. 

How and where exactly the three virion bricks are brought together for assembly is still a controversial issue. The junction between ER and LDs probably forms the site for virion assembly [85,132]. The apposition of the two organelles could happen early during the LD biogenesis and involve nascent LDs, or depend on the LD back-trafficking towards the ER [133]. The localization of core is however a more subtle process, as it depends both on p7 and NS2 expression [134]. An ER rather than a LD accumulation of core, characterizing high-titer- (Jc1) and low-titer- (JFH1) viruses respectively, favors HCV production. This suggests that core needs to be recruited back from the LD surface to the ER vicinity [134]. The interaction between NS2 and the NS3/4A complex also contributes, directly or not, to core unloading from the LDs [86]. In addition to the viral non-structural proteins, the AP2M1 host factor, a component of the clathrin adaptor protein complex 2, seems to be recruited by core at the LD surface and necessary for core’s journey back to the ER [135]. The point of sending core onto LDs and bringing it back to the ER vicinity is possibly to recruit LDs and thereby bring the VLDL biogenesis machinery close to the virion factory. 

This is getting even more complicated when trying to integrate the viral RNA into this schema. Four viral proteins have been shown to interact with HCV RNA: core, NS5A, the NS3/4A helicase domain and the polymerase NS5B [20]. NS5A and/or NS3/4A might transfer newly replicated RNA genomes in the assembly sites, while core interaction with the viral RNA enables nucleocapsid assembly. Interestingly, NS5A interacts with core and with LDs [85,136,137]. Its phosphorylation status may tune the delicate balance between the two RNA functions: template for replication and scaffold for assembly [136,138]. Whether NS5A acts by anchoring LDs at the replication complex (RC) proximity or by actively transporting the viral RNA at the LD surface before the recruitment of the LD back to the ER is unclear. NS3/4A on the other hand, might also bring together newly replicated HCV RNA and envelope glycoproteins, via NS2 [86,128,139], and/or via NS3/4A interaction with core [140,141].

Although the glycoproteins are anchored in the ER membrane, the connection with the other virion components might be direct or indirect. An interaction between core and E1 has been reported *in vitro* [142,143,144] and depends on core oligomerization [144]. This interaction has however not been validated in an infectious system and is challenged by core and E1 topologies. Alternatively, NS2 might serve as a mediator as it interacts both with E1 and E2 [139,145,146], with the RCs via NS5A [145,146] or NS3-A4 [86,139,146], and with p7 [84,132,145,146], another assembly specialist. Importantly, p7 is necessary for the building of E1E2-NS2-NS3/4A complexes around NS2, and therefore participates in the connection between HCV envelope proteins and RC-nascent genomic RNA, and possibly RNA-bound core protein [139]. It also colocalizes perfectly with E2 in HCV‑infected cells [84]. Moreover, although no physical interaction has been proven yet, genetic interactions and the capacity of p7 to determine core localization further suggest that p7 might bridge NS2 and core protein [134,147]. Altogether, core (possibly recruited by p7) and the envelope glycoproteins (recruited by NS2) could be brought together by the NS2-p7 interaction [132,145,146], while the viral RNA might be recruited to core via NS5A [136,138] or to the assembly site via NS3/4A [86,139]. 

**Figure 7:**
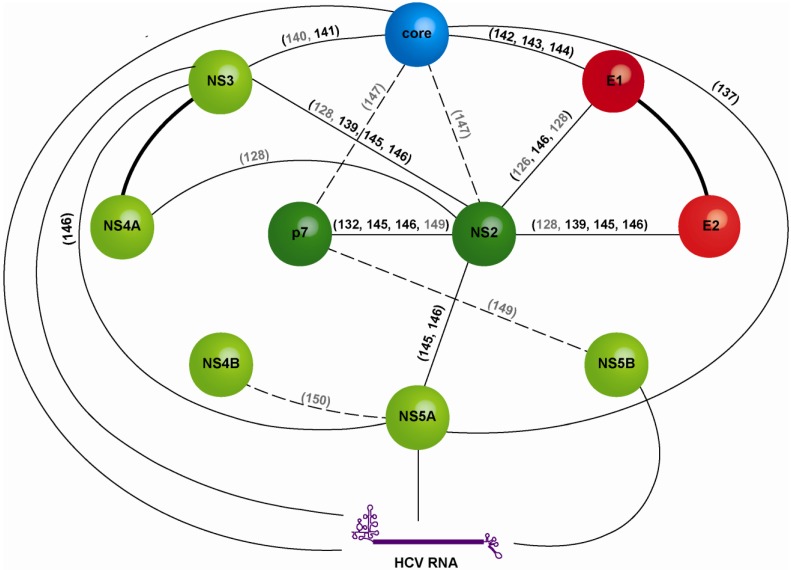
Network of interactions between HCV virion components and non-structural proteins that support HCV assembly. The diagram is based on physical (continuous arrows, references in black) and genetic interaction data (dashed arrows, references in grey). Thick black lines stand for stable, long-lived interactions that are supported by multiple lines of evidence. The color code for the individual proteins is similar as in Figure 1 and Figure 6. The detailed characterization of the individual interactions (expression system, direct or indirect interaction, *etc.*) can be found in the original articles.

The link between structural and non-structural proteins made by p7 and NS2 has been extensively explored. It is supported by the position of their precursor in the polyprotein and by the necessary compatibility between the protein sequences. Thus, the capacity of an HCV chimera to produce high-titer virus depends on the compatibility between NS2 first TM segment and the structural proteins on one side, and between NS2 C-terminus and the non-structural proteins on the other side [148]. Further protein sequence compatibilities have been evidenced by the emergence of second-site mutations arising in assembly-impaired HCV mutants [126,128,132,137,139,140,141,142,143,144,145,146,147,149,150,151]. Such genetic interactions are indicative of direct or indirect physical interactions between the proteins in the assembly process. Figure 7 attempts to summarize the complex network of interactions supported by these genetic studies and physical interaction data. 

Once the virion components are gathered at the assembly site, virion morphogenesis takes place with genome encapsidation and capsid envelopment at the ER membrane. A panel of assays is available to pinpoint a block in this assembly sequence in the context of assembly-deficient virus mutants or host cell perturbations [152]. Note that no encapsidation signal has been reported for HCV and the core protein seems to be able to coat any structured RNA [153,154]. The specificity of HCV encapsidation is probably a consequence of the spatial proximity of the capsid protein and newly replicated RNA genomes [87,155] and of the interaction between core protein and replication complexes. While capsid formation was described to occur upon expression of core protein alone [154], naked capsid have not been observed in HCV-infected cells, suggesting that encapsidation completion is concomitant with envelopment. The global role of the envelope glycoproteins in capsid formation and envelopment is still a matter of debate, but it seems that HCV mutants deleted of the E1E2-coding regions form intact nucleocapsids [140,156] but are not enveloped [152]. Moreover, chimeric viruses carrying glycoproteins derived from a genotype different from the remaining viral proteins, are arrested at the step of capsid envelopment [157]. This indicates that the interplay between E1E2 and other viral factors is genotype-dependent and required for proper envelopment of viral capsids. 

### 3.3. E1E2 Determinants for HCV Assembly

Thanks to the combination of HCVcc, HCVpp and HCV_TCP_ systems, it is possible to distinguish the effect of E1E2 variation on virion assembly, from its effect on entry. First of all, the expression as part of the common polyprotein precursor is neither crucial for entry nor for assembly. Indeed, glycoproteins expressed in *trans*, in a single or two separate cistrons, are incorporated into infectious HCVpp [27,33] and HCV_TCP_ [34,102,106,108,109,158]. 

Secondly and as expected, folding and glycosylation of E1 and E2, two tightly linked processes [50,54] determine not only entry [50,79] but also assembly efficiency. This is evidenced in the HCVcc system by the mutation of particular E1E2 glycosylation sites [79] or the inhibition of ER-glucosidases with iminosugar derivatives [159]. 

Thirdly, E1E2 heterodimerization is a prerequisite for E1E2 to mediate entry [42], but its importance for virion assembly is unclear. Indeed, an E1 TMD mutant, unable to heterodimerize upon ectopic expression of genotype 1a glycoproteins [32], could, in the context of the Jc1 virus, complete capsid envelopment and secrete virions as efficiently as the wild type, although with decreased specific infectivity [152]. The capacity of this E1 mutant to dimerize with E2 in the Jc1 context remains to be addressed. However, it is possible that the density of E1E2 proteins at the assembly site and their possible cross-linking by disulfide bonds upon virus budding [10] replaces the need for E1E2 heterodimerization during assembly. 

Moreover, compatibility within E1E2 sequences and between E1E2 and other viral proteins determines the assembly success. By switching the genotype or subtype of one of the two glycoproteins in the HCVpp or HCVcc systems, several interfaces between E1 and E2 were described as important for virion entry or assembly [27,28,30]. Interactions between domains within the E2 ectodomain were also identified, for instance with the role of HVR2 and IgVR in HCVcc assembly [27,47]. Moreover, switching the genotype of both glycoproteins within the full-length genome abrogated capsid envelopment [157] despite proper localization of E1E2, thus strengthening the importance of a crosstalk between E1E2 and other viral proteins, as discussed before and illustrated in Figure 7. Finally, variations in the glycoprotein sequence can also affect the quality of the virion (see Section 3.5), suggesting a necessary compatibility between glycoproteins and host proteins.

### 3.4. The Driving Force for Virion Budding

**Figure 8:**
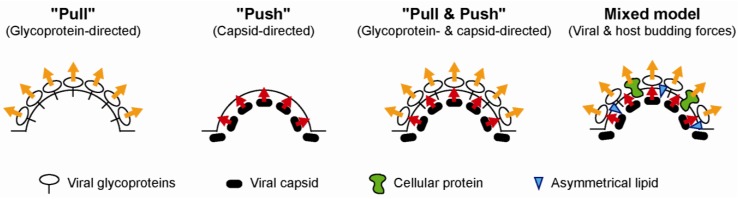
Pull or push, or pull and push: Which driving force for HCV budding?

Enveloped viruses hijack a portion of cellular membrane for envelopment of the viral capsid. In the case of HCV, the virions bud in the ER lumen and are released through the secretory pathway. For budding to happen, curvature and scission of the host membrane are needed. Enveloped viruses have evolved three main strategies to induce the membrane curvature and initiate budding [95] (Figure 8). In the “push” model, the virion inside, that is to say the capsid or matrix, exerts a pressure on the membrane. In the “pull” model on the contrary, the budding driving force comes from the outside, as the glycoproteins pull the membrane. The third model, “push and pull” combines the two forces, and can involve the direct recruitment of the nucleocapsid or matrix by the envelope proteins. 

It is still unclear which model applies to HCV budding, although several hints favor the preponderant role of the glycoproteins in the process. Although in heterologous expression systems, core protein can oligomerize and drive envelopment without the need for any other viral protein [160,161], in the HCVcc system however, clones deleted of HCV envelope proteins release much less core protein than full-length clones [148]. Most importantly, we showed that deletion of the glycoprotein coding sequence or replacement by glycoprotein genes from another genotype abolishes HCV capsid envelopment [152,157]. P7 mutants exhibit a similar envelopment defect, along with an incomplete or aberrant capsid assembly [152]. These concomitant defects further suggest that capsid envelopment might be a prerequisite for the completion of the capsid assembly. More precisely, the membrane curvature induced by virus budding might provide a favorable environment for the capsid oligomerization and RNA encapsidation due to the affinity of core domain 2 for membranes.

Moreover, the incorporation of E1E2 as large covalent complexes at the virion surface [10] is another argument in favor of the “pull” mechanism. A more precise characterization of these complexes, regarding their size and composition, would be of great interest. One hypothesis is that HCV glycoproteins form a cage around the virion. The related flaviviruses and, more globally, viruses with a class II fusion protein exhibit such a glycoprotein lattice at their surface, which is the main budding force [73]. However, the rather heterogeneous and unstructured nature of cell free HCVcc particles and the difficulty to detect the glycoproteins on their surface [14] cast some doubt that this is also true for HCV. Yet, the presence of E1E2 covalent complexes larger than 400 kDa at the virion surface implies a number of strong lateral interactions between envelope glycoproteins, which could facilitate their pulling force and might suffice to induce a membrane curvature. The situation is different for HCVpp, whose glycoproteins might not need to form covalent high-order complexes [10,36], since their budding is triggered by the retroviral machinery.

On a different note, the importance of the capsid in the budding process is undermined by the extreme cases found among HCV relatives. For example, CSFV (classical swine fever virus), a pestivirus, can still assemble fully infectious virions after the deletion of its capsid-coding sequence [162]. Furthermore, GB viruses type A and C (GBV-A and GBV-C) naturally do not encode a nucleocapsid [163]. 

The capacity of viral envelope glycoproteins to produce subviral particles can be a further indication for their ability to trigger budding. Importantly, such subviral particles, decorated with envelope proteins but lacking nucleocapsids, have been reported for flaviviruses [164,165]. In the case of HCV, the production of subviral particles has been described upon expression of E1 and E2 in Caco-2, HepG2 or Huh-7 cell lines [166]. Whether the expression of HCV glycoproteins alone recapitulates the authentic intracellular trafficking and behavior of E1E2 is unclear and the release of subviral particles in cells producing HCVcc has not been reported yet. Similar capsid-free E1E2‑positive particles have however also been detected in patients in a large excess as compared to low-density infectious particles [167]. These empty subviral particles have a low density comparable to VLDL and, when generated in Caco-2 or HepG2 cell lines, are associated with apoB. However, no subviral particles are secreted upon E1E2 expression in the HeLa cell line which lacks the ability to produce lipoproteins [166]. Altogether and to sum things up, several pieces of evidence suggest that in this system, the budding was triggered by the lipoprotein biogenesis rather than by E1E2 alone: (i) E1E2-positive structures have a low density, (ii) they associate with apoB in some cell lines and in most patients and (iii) they are not secreted from cell lines lacking the lipoprotein assembly machinery. 

Finally, other motors could drive HCV budding [95]. First of all, as mentioned above, HCV could use the lipoprotein biogenesis machinery for its own budding, therefore involving host factors as driving engines rather than its structural proteins. Like HCV, VLDL initiates its assembly in the ER. The formation of VLDL is concomitant with apoB lipidation. Our results and others however indicate that apoB is not necessary for HCV assembly and release [119,123,124], casting a doubt on this hypothesis, at least for the cell culture scenario. The HCV and lipoprotein assembly pathways seem only to connect after the virus envelopment [124], which is also more intuitive given that VLDL precursors bud with a membrane monolayer only. Finally, the recruitment of specific asymmetrical lipids at the site of assembly might facilitate or induce a membrane curvature conducive to budding. This could be a cue for the role of the PLA2G4A phospholipase and its arachidonic acid product in HCV assembly [168]. 

Completion of virion budding requires scission of the virion from the host cell membrane. The ESCRT machinery is used by many viruses to fulfil this function [95] and might also be involved in HCV production [158,169,170]. So far however, only subsets of the ESCRT machinery have been associated with HCV production and it remains unclear whether they contribute to HCV scission, trafficking or simply indirectly affect HCV production by perturbation of the host membrane composition [158]. 

### 3.5. Participation of the Host Lipoprotein Machinery in HCV Assembly and Entry Process

Several lines of evidence from different laboratories highlight that the lipoprotein machinery is crucial for HCV assembly [118,119,123,124,171,172]. Of note, very recent evidence indicates that apoE alone is the minimal essential lipoprotein required for production of infectious HCV particles [119,123,124]. So far no clear differences between HCVcc particles originating from Huh‑7.5 cells or non-liver cells expressing apoE were found with regard to density and neutralization by glycoprotein or receptor-specific antibodies. However, it is striking that virions released from the latter cells have a ca. 5-fold lower specific infectivity [124], which may be related to the incorporation of a limited spectrum of lipoproteins. In that regard it is interesting to note that immunolabeling of highly purified HCVcc particles from Huh-7.5 cells recently revealed that not only apoE but also apoA1 and apoB are incorporated [14]. Since apoA1 and apoB are lacking in the non-liver cells, lack of these factors may reduce the infectiousness of HCVcc released from these cells. Alternatively or in addition, the atypical lipid composition of the virus released from Huh-7.5 cells, enriched in cholesterol esters thus resembling VLDL [16], may not occur in viruses produced in non-liver cells. In addition to this, HCV from primary human liver cells cultured *in vitro* and/or from humanized animals has been shown to display a lower density and higher specific infectivity compared to Huh-7.5-derived HCV [99,100]. Comparative analysis of the protein and lipid content of viruses derived from all these different sources combined with functional cell entry assays should help to define the natural lipoprotein coat of HCV including its relevance for cell entry and infection.

Besides, how and when exactly lipoprotein and HCV syntheses converge to release a lipoviroparticle is unclear. A possible scenario is illustrated in Figure 6 (right side of the figure). Briefly, dense immature intracellular HCV particles [117] might mimic the lipoprotein precursors [173] and undergo a similar maturation process. This maturation may involve the fusion with a luminal LD and concomitant acquisition of the exchangeable apoE [101]. This would result in the release of mixed lipoviroparticles with lower density and larger size [117]. This model is consistent with our recent data indicating that apoE controls a post-envelopment stage of virus production [124]. The use of LDs as a platform for HCV morphogenesis might be sufficient to bring together lipoprotein and HCV assembly factories. Alternatively, specific interaction between HCV proteins and apolipoproteins could help tighten the link at an early stage. One study reported the interaction of E1E2 with serum lipoproteins *in vitro* [174], and preliminary data from others suggested an interaction between E2 and apoE [175]. ApoAI was shown to bind NS5A [78]. Finally, an interaction between NS5A and apoE has repeatedly been described [119,176,177,178] and might contribute to HCV assembly [176,178]. Given the topology and subcellular localization of the two proteins, it would however be interesting to confirm this interaction in live cells. 

The complexity of the HCV outer coat with its incorporation of lipoprotein component is partly responsible for the extremely complicated entry sequence (for a review, see [7]). Lipoproteins and HCV particles indeed share a number of attachment or entry factors (glycosaminoglycans, LDL receptor and SR-BI), that add up to the already long list of HCV-specific entry factors. It is possible therefore that the lipoprotein moiety of the virion plays a major role in the initial attachment of the virion onto the target cells, before the less exposed viral glycoproteins can access their receptors. Consistently, antibodies targeting apoE neutralize HCVcc infection [118,119], at the binding stage [179,180]. It is finally also possible that the lipid cargo of the lipoviroparticle facilitates E1E2‑mediated virus fusion by locally modifying the lipid composition and fluidity of the host cell membrane. Moreover, incorporated lipoproteins may directly influence the conformation and function of E1E2 during HCV cell entry. Of note, apoC1 was shown to enhance HCVcc and HCVpp infection by increasing the fusion rate of these particles. This phenotype was modulated by the hypervariable region 1 at the N-terminus of E2 [181], which is in principle consistent with the notion that apoC1 may influence the function of this subdomain. Clearly more work is needed to differentiate direct effects of virion-associated lipoproteins on HCV cell entry from indirect influences on glycoprotein function.

Finally, glycoprotein determinants modulate the virion-lipoprotein association. This is particularly evident with E2 HVR1. Indeed, the deletion of HVR1 results in a decrease in the number of very-low-density particles [182] and in their loss of infectivity [182,183], whereas intermediate density fractions are indistinguishable from the wild type control in term of number of particles and specific infectivity [182,183]. Removal of HVR1 also increased the exposure of the CD81 binding site and neutralization by specific antibodies [182,183,184]. Intriguingly, a point mutation in E2 or mutation of individual cysteine residues in E1 can recapitulate several of these features [58,185]. 

## 4. Exposure of the E1E2 Functional Complex at the Virion Surface

### 4.1. Travelling Incognito: The Virion with Lipoprotein and Glycan Masks on

In order to establish and maintain lifelong chronic infections in immunocompetent hosts, HCV glycoproteins have likely been shaped to optimize entry efficiency and at the same time neutralization escape [80]. Thus, immunogold labeling or immunocapture of HCVcc is always more efficient with anti-apoE than with anti-E2 antibodies [14,16], suggesting a very discrete exposure of the glycoproteins.

The variability of the E1E2 sequence reflects the strong immune pressure exerted on these proteins and the remarkable flexibility to escape these antibodies while preserving essential functions for cell entry. The maturation of the glycoproteins, the way they are incorporated in the virion and the virion association with lipoproteins constitute additional factors that permit viral escape from humoral immunity. First of all, the presence of a dense glycan shield on both glycoproteins renders the whole glycoprotein complex globally less immunogenic and hides the receptor-binding sites [79,80]. E2 contains variable regions that are functionally highly flexible thus tolerating sequence variation. Their exposure at the E2 periphery [62] favors them as immunodominant regions and drives the immune response away from more important receptor-binding sites. Moreover, HVR1 seems to physically conceal conserved viral domains involved in CD81 interaction since its deletion enhanced E2 binding to soluble CD81 and much increased virus neutralization by patient sera and a panel of monoclonal antibodies [182,183]. Then, the organization of E1E2 in heterodimers, themselves engaged in large covalent complexes might also decrease the exposed protein surface [10]. Finally, the virus association with lipoproteins further shields the virion and the surface glycoproteins. The connection between HVR1, lipoprotein association and antibody-mediated neutralization and the correlation between HCV density and neutralization efficiency underline the propensity of the lipoprotein part of the lipoviroparticle to hide the glycoprotein complexes [182,185]. 

The influence of all these factors differ between HCVpp and HCVcc (different glycan maturation, oligomerization, lipoprotein association, number of E1E2 copies per virion) [10,36]. This may result in differences in antibody neutralization and entry efficiencies between the two systems. However, the comparative data on this topic are sparse and contradictory [186,187,188,189,190]. Also, the quality of the HCV‑associated lipoproteins certainly differs depending on the host system used to produce HCVcc. Thus, despite many similarities (e.g., density profile, CD81-, E2- and apoE-specific neutralization patterns), virions produced in 293T cells supplemented with apoE exhibit a lower specific infectivity than those secreted by Huh7-derived cells [124]. The cause for such a discrepancy (e.g., lipoprotein, lipid or viral factor) remains to be determined.

### 4.2. E1E2 Oligomerization at the Virion Surface

Although the intracellular unit for HCV glycoprotein is the E1E2 heterodimer, surprisingly, a majority of large (>400 kDa) covalent-bound heterodimers are incorporated on the virion [10]. Importantly, the large glycoprotein complexes differ from the covalent-bound aggregates detected upon heterologous E1E2 expression since they retain a functional conformation [10]. Importantly, E1E2 oligomerization is different on HCVpp. In HCVpp-producing cells, both functional heterodimers and non-functional aggregates are detected, the earliest being specifically incorporated in the virion [36,37].

The discrepancy between intra- and extracellular E1E2 oligomerization status in the HCVcc system suggests a late rearrangement of the glycoproteins, possibly triggering the budding process (see Section 3.4 and Figure 8). The conversion between non-covalent heterodimers and large covalent complexes might coincide with the reshuffling of existing intramolecular disulfide bonds. Alternatively, disulfide bonds might be formed *de novo* between previously buried free cysteine residues that would become exposed on the glycoprotein surface upon a change in protein conformation. However, the motor driving this rearrangement of the disulfide bonds is unknown. The first hypothesis could involve the proximity or recruitment of a disulfide isomerase. The second scenario could be triggered by a local redox or electric charge perturbation. 

The nature of the covalent envelope complexes remains enigmatic. However, our most recent data suggest a hierarchical organization of E1E2, first in heterodimers, themselves bound into E1-driven trimers, which are finally clustered with disulfides [23]. Whether cellular proteins, such as the apolipoproteins, are part of the largest complexes is yet unknown.

### 4.3. From Assembly to Entry: The Issue of Packaging an Entry-Competent Glycoprotein Complex

Viral envelope proteins should be efficiently packaged into the virion, in a way that they are competent for mediating viral fusion with naive cells, yet do not induce premature fusion with the producer cell upon virion secretion. Viruses from the *Flaviviridae* family have evolved different solutions to solve this problem. Flaviviruses incoporate an immature form of the prM companion protein that needs to be cleaved for the fusion protein E to be entry competent. This cleavage is mediated by a furin protease in the secretory pathway [74]. Pestiviruses, on the other hand, incorporate disulfide-linked glycoprotein heterodimers that require disulfide isomerisation to become fusion‑competent [191]. 

**Figure 9:**
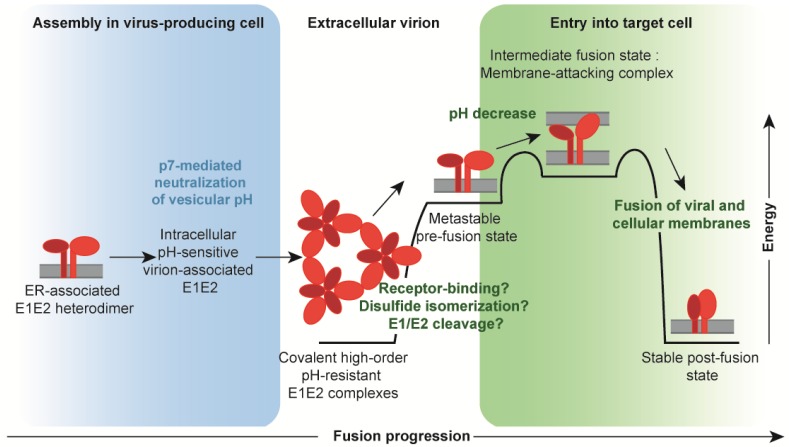
Getting ready for fusion. Hypothetical model of E1E2 rearrangements necessary to mediate viral fusion. Upon virion assembly or release, E1E2 heterodimers reorganize into large covalently bound complexes. At the extracellular virion surface, E1E2 complexes are pH-resistant and need a priming event before they become susceptible to low pH and fusion-competent (CD81 priming [192], possibly other triggers). This could occur concomitantly with a dissociation of the large E1E2 complexes, although a demonstration for this is lacking. In early endosomes, pH acidification will trigger the exposure of the fusion peptide so that viral and host membranes can merge and the fusion protein(s) finally reaches its low-energy post-fusion state. Note that in absence of structural data on E1 and E2 proteins, the identity of the fusion protein was left open on the schema.

In the case of HCV, the glycoprotein maturation step seems to occur upon entry rather than assembly, since, contrary to flaviviruses, the secreted virions are still pH-resistant, and therefore not yet competent to mediate fusion (Figure 9). Only upon cell binding, low pH-dependent fusion can be triggered in a time- and temperature-dependent manner [13]. What happens during this time lapse is still not fully clear. However, recent evidence suggested that the interaction with CD81 may trigger a conformational change permitting low pH-induced membrane fusion [192]. Moreover, in the case of HCV, neither E1 nor E2 is cleaved during secretion [10]. A cleavage during virus entry, as is the case for the *Filoviridae* [193], remains an option that so far has not been proven or disproven. 

Isomerisation of the disulfide bonds as observed for pestiviruses is an appealing possibility for HCV, which incorporates disulfide-linked glycoprotein complexes [10]. It is therefore possible that E1E2 cross-linking by disulfide bonds replaces the need for a glycoprotein cleavage by keeping the virion fusion-incompetent until it reaches a plasma membrane- or endosome-associated isomerase. This model is also consistent with the pH-sensitivity of HCVpp whose glycoproteins are not covalently linked, and which do not require priming before pH-triggered fusion at the plasma membrane [36]. Alternatively, as demonstrated for some retroviruses [194] and as mentioned above, receptor interaction might prime HCV glycoproteins for later fusion in early endosomes. Indeed, virus pre‑treatment with a soluble form of the CD81 receptor renders it permissive for low-pH-triggered fusion at the plasma membrane [192]. 

Finally, while HCV envelope proteins are pH-resistant once secreted, a study reported that this might not yet be the case during virion egress [195]. This is somehow counter-intuitive because the selective pressure to harbor pH-resistant glycoproteins should act during the virion trafficking through the acidic secretory vesicles rather than after release. Indeed, outside the cell, the pH is close to neutral and pH-resistance constitutes an additional barrier to overcome for successful entry. However, the authors of this study showed that p7 addressing to post-ER compartments of the secretory pathway could equilibrate the pH in this organelle and thereby protect the viral particle on the way out. 

The way HCV glycoproteins interact with their host receptors and mediate virus entry has been reviewed by others [7]. While E2 fulfills this task, recent evidence suggest that it is assisted in it by the companion E1 protein. Indeed, as mentioned above, the fusion task has not yet been firmly assigned to E1 or E2, and although E1 might not directly interact with any virus receptors, recent evidence suggest that E1 modulates E2 binding to CD81 [58]. Furthermore, albeit rare, some antibodies directed against E1 can neutralize HCV infectivity [148,196,197,198]. Therefore, even simple questions regarding E1E2 role and incorporation in the virion remain unanswered and still arouse interest into the field of HCV glycoprotein research. 

## 5. Conclusions and Future Challenges

The recent acceleration into HCV assembly research has highlighted a complicated but concerted process involving every single HCV protein plus a still underestimated number of host factors. Integrating, in time and space, the numerous protein-protein interactions and assembly cofactors described is a future challenge to take up for a more comprehensive view on the HCV assembly complexes and their coordination. The difficulty to image assembly events has led to the optimization of indirect assays to evaluate the different steps of assembly, including virus envelopment and the incorporation of E1 and E2 glycoproteins. Proteomics analysis of assembly complexes and the development of new tools to track assembly cofactors in live cell might yield a broader picture. Larger‑scale purification of the virion-embedded glycoprotein complexes will help understanding the organization of the virion outer shell, how large complexes can be built from heterodimers and why they might need priming for entry. Similarly to assembly, HCV entry is extremely complex and the role of the multiple host factors must now be ordinated and correlated with virion progression in the host cell but also with changes in HCV envelope shell and glycoprotein complexes. As long as the structure of the E1E2 glycoprotein complex remains unknown, grey areas will shade our understanding of the HCV entry and assembly. Structural and functional studies on other *Flaviviridae* have focused on the E2 homologs, but the role of E1 in HCV biology might deserve more attention. After all, it is still unclear which one of E1 or E2 protein mediates fusion or whether both glycoproteins cooperate for this process. Ultimately it is to be hoped that increased understanding of the function(s) of the E1 and E2 proteins combined with structural information will guide the way to develop immunogenic antigens that may be useful components of future vaccine formulations against hepatitis C. 
